# Synthetic lung surfactants containing SP-B and SP-C peptides plus novel phospholipase-resistant lipids or glycerophospholipids

**DOI:** 10.7717/peerj.2635

**Published:** 2016-10-27

**Authors:** Robert H. Notter, Rohun Gupta, Adrian L. Schwan, Zhengdong Wang, Mohanad Gh Shkoor, Frans J. Walther

**Affiliations:** 1 Department of Pediatrics, University of Rochester, Rochester, NY, United States; 2 Department of Pediatrics, Los Angeles Biomedical Research Institute at Harbor-UCLA Medical Center, Torrance, CA, United States; 3 Department of Chemistry, University of Guelph, Guelph, Ontario, Canada; 4 Department of Pediatrics, David Geffen School of Medicine, University of California, Los Angeles, Los Angeles, CA, United States

**Keywords:** Phospholipase-resistant lung surfactants, Super Mini-B DATK, SP-B and SP-C peptide mimics, Surfactant protein B (SP-B), SP-Css ion-lock 1, DEPN-8, Synthetic lung surfactant, PG-1, Surfactant protein C (SP-C), Acute respiratory distress syndrome (ARDS)

## Abstract

**Background:**

This study examines the biophysical and preclinical pulmonary activity of synthetic lung surfactants containing novel phospholipase-resistant phosphonolipids or synthetic glycerophospholipids combined with Super Mini-B (S-MB) DATK and/or SP-Css ion-lock 1 peptides that replicate the functional biophysics of surfactant proteins (SP)-B and SP-C. Phospholipase-resistant phosphonolipids used in synthetic surfactants are DEPN-8 and PG-1, molecular analogs of dipalmitoyl phosphatidylcholine (DPPC) and palmitoyl-oleoyl phosphatidylglycerol (POPG), while glycerophospholipids used are active lipid components of native surfactant (DPPC:POPC:POPG 5:3:2 by weight). The objective of the work is to test whether these novel lipid/peptide synthetic surfactants have favorable preclinical activity (biophysical, pulmonary) for therapeutic use in reversing surfactant deficiency or dysfunction in lung disease or injury.

**Methods:**

Surface activity of synthetic lipid/peptide surfactants was assessed *in vitro* at 37 °C by measuring adsorption in a stirred subphase apparatus and dynamic surface tension lowering in pulsating and captive bubble surfactometers. Shear viscosity was measured as a function of shear rate on a Wells-Brookfield micro-viscometer. *In vivo* pulmonary activity was determined by measuring lung function (arterial oxygenation, dynamic lung compliance) in ventilated rats and rabbits with surfactant deficiency/dysfunction induced by saline lavage to lower arterial PO_2_ to <100 mmHg, consistent with clinical acute respiratory distress syndrome (ARDS).

**Results:**

Synthetic surfactants containing 5:3:2 DPPC:POPC:POPG or 9:1 DEPN-8:PG-1 combined with 3% (by wt) of S-MB DATK, 3% SP-Css ion-lock 1, or 1.5% each of both peptides all adsorbed rapidly to low equilibrium surface tensions and also reduced surface tension to ≤1 mN/m under dynamic compression at 37 °C. However, dual-peptide surfactants containing 1.5% S-MB DATK + 1.5% SP-Css ion-lock 1 combined with 9:1 DEPN-8:PG-1 or 5:3:2 DPPC:POPC:POPG had the greatest *in vivo* activity in improving arterial oxygenation and dynamic lung compliance in ventilated animals with ARDS. Saline dispersions of these dual-peptide synthetic surfactants were also found to have shear viscosities comparable to or below those of current animal-derived surfactant drugs, supporting their potential ease of deliverability by instillation in future clinical applications.

**Discussion:**

Our findings support the potential of dual-peptide synthetic lipid/peptide surfactants containing S-MB DATK + SP-Css ion-lock 1 for treating diseases of surfactant deficiency or dysfunction. Moreover, phospholipase-resistant dual-peptide surfactants containing DEPN-8/PG-1 may have particular applications in treating direct forms of ARDS where endogenous phospholipases are present in the lungs.

## Introduction

Therapy with exogenous surfactant drugs has proven to be life-saving in preventing or treating respiratory distress syndrome (RDS) in preterm infants (Notter, 2000; Polin et al., 2014), and extensive research has been directed at extending this intervention to pediatric and adult patients with acute respiratory distress syndrome (ARDS) (Raghavendran, Willson & Notter, 2011; Zhang et al., 2013). However, surfactant therapy in ARDS is challenging, and to date has not been found to improve mortality in clinical trials in adult patients. The lack of success of surfactant therapy in adults with ARDS likely reflects multiple factors, including differences in lung injury etiology, variations in the activity and inhibition resistance of lung surfactant drugs, differences in administration and ventilation protocols, and the difficulty of delivering adequate exogenous surfactant concentrations to the alveoli following tracheal instillation in patients with injured, inflamed lungs (Raghavendran, Willson & Notter, 2011; Albert, 2012). Despite this, there remains a strong scientific rationale for the potential efficacy of surfactant therapy in direct pulmonary forms of ARDS based on a mechanistic knowledge of surfactant dysfunction and the documented ability to reverse this phenomenon *in vitro* and in animal models (Wang et al., 2005; Raghavendran, Willson & Notter, 2011; Albert, 2012). This paper reports new synthetic lipid/peptide lung surfactants of high activity that may have future utility not only for treating RDS in infants, but also for improving surfactant therapy for direct pulmonary forms of ARDS that continue to have substantial levels of patient mortality.

One potentially detrimental factor for the efficacy of surfactant therapy in ARDS is that glycerophospholipids, the major compositional constituents by weight in all current clinical lung surfactant drugs, are sensitive to chemical degradation by phospholipases present in increased concentrations in the pulmonary interstitium or alveoli during inflammatory lung injury (Notter, 2000; Notter et al., 2007). Phospholipase degradation of exogenous surfactant phospholipids has the potential not only to reduce active drug concentration, but also to generate byproducts such as free fatty acids and lysophosphatidylcholine that are themselves severe biophysical inhibitors of surfactant activity (Wang & Notter, 1998; Holm, Wang & Notter, 1999; Hite et al., 2012; De Luca et al., 2013). We have previously reported the synthesis of active analogs of lung surfactant glycerophospholipids that have specific molecular substitutions to confer chemical resistance to phospholipase degradation (Turcotte et al., 1991; Wang et al., 2003; Notter et al., 2007; Schwan et al., 2011). The current study uses two of these phospholipase-resistant lipids, DEPN-8 and PG-1, as components in new synthetic lipid/peptide surfactants.

DEPN-8 is a C16:0 diether phosphonolipid analog of dipalmitoyl phosphatidylcholine (DPPC) (Turcotte et al., 1991; Wang et al., 2003), and PG-1 is a C16:0:C16:1 ether-linked phosphonolipid analog of palmitoyl-oleoyl phosphatidylglycerol (POPG) (Schwan et al., 2011), the major class of anionic phospholipids in native surfactant. Prior work by our group has shown that synthetic surfactants containing DEPN-8 or 9:1 DEPN-8:PG-1 plus an active surfactant protein (SP)-B peptide (Mini-B or Super Mini-B) have significant surface activity (Walther et al., 2007; Schwan et al., 2011). This earlier work is extended here to synthetic lipid/peptide lung surfactants containing recently reported active mimics of hydrophobic surfactant apoproteins, i.e. Super Mini-B (S-MB) DATK and SP-Css ion-lock 1 peptides.

S-MB DATK (41 residues) incorporates the functional helix-turn-helix saposin fold structure of human SP-B, along with its functionally important N-terminal insertion sequence (Walther et al., 2010; Notter, Wang & Walther, 2016). This peptide also incorporates a designer-loop DATK substitution to improve molecular stability and ease of folding relative to the parent S-MB molecule (Walther et al., 2013; Notter, Wang & Walther, 2016). S-MB DATK is quite resistant against inactivation by serum albumin leaking into the airways during lung injury and the formation of C16:0 lysophosphatidylcholine and release of free fatty acids through breakdown of glycerophospholipids by phosphorylases (Notter, Wang & Walther, 2016). SP-Css ion-lock 1 (34 residues) closely mimics the amino acid sequence of human SP-C, but with a stabilizing ion-lock (salt-bridge) between residues E(20)–K(24) to maintain an active helical structure and minimize detrimental non-specific beta (amyloid-like) formation (Walther et al., 2013; Walther et al., 2014). We have recently reported the high activity of single-peptide synthetic surfactants containing glycerophospholipids combined with either S-MB DATK (Notter, Wang & Walther, 2016) or SP-Css ion-lock 1 (Walther et al., 2014) alone. A major focus in the current study is on the high surface and pulmonary activity of dual-peptide synthetic surfactants containing both peptides, as was first demonstrated in our collaboration with the group of Curstedt (Almlén et al., 2010). These dual-peptide synthetic surfactants containing either phospholipase-resistant lipids (9:1 DEPN-8:PG-1) or glycerophospholipids (5:3:2 DPPC:POPC:POPG) are shown to have greater efficacy in improving oxygenation and compliance in mechanically ventilated animals with ARDS compared to analogous single-peptide surfactants.

## Materials and Methods

### Synthetic peptides

**Figure 1:**
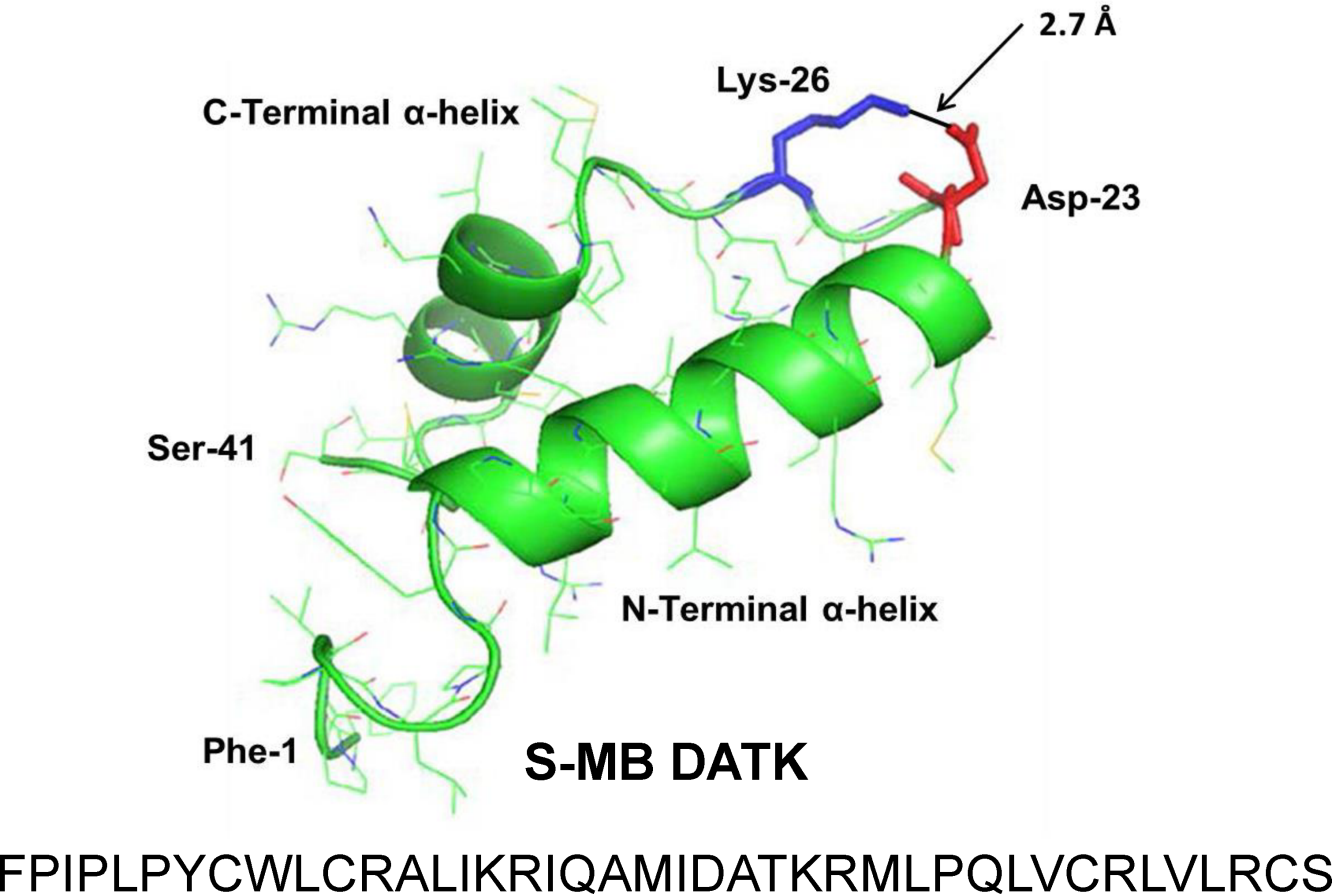
Linear sequence and homology-modeled ribbon structure of Super Mini-B (S-MB) DATK peptide. The figure shows the homology modeled structure of the 41 residue sequence of S-MB DATK (Notter, Wang & Walther, 2016). This active mimic of human SP-B contains its functional amphipathic N- and C-terminal helices, lipophilic insertion sequence, and primary saposin fold character plus a structure-stabilizing DATK substitution. Helical residues in the homology modeled peptide structure are highlighted in green ribbon, with disordered and loop-turn segments represented as green tubes. The turn-stabilizing ion-pair is rendered in red (Asp^−^-23) and blue (Lys^+^-26), with a calculated equilibrium separation distance of 2.7 Å. The characteristic “saposin fold” of reduced S-MB DATK encompasses the N-terminal *α*-helix (residues 8–21), the loop-turn (residues 22–29) and C-terminal *α*-helix (residues 30–37), while the additional lipophilic N-terminal insertion sequence includes residues 1–7.

**Figure 2:**
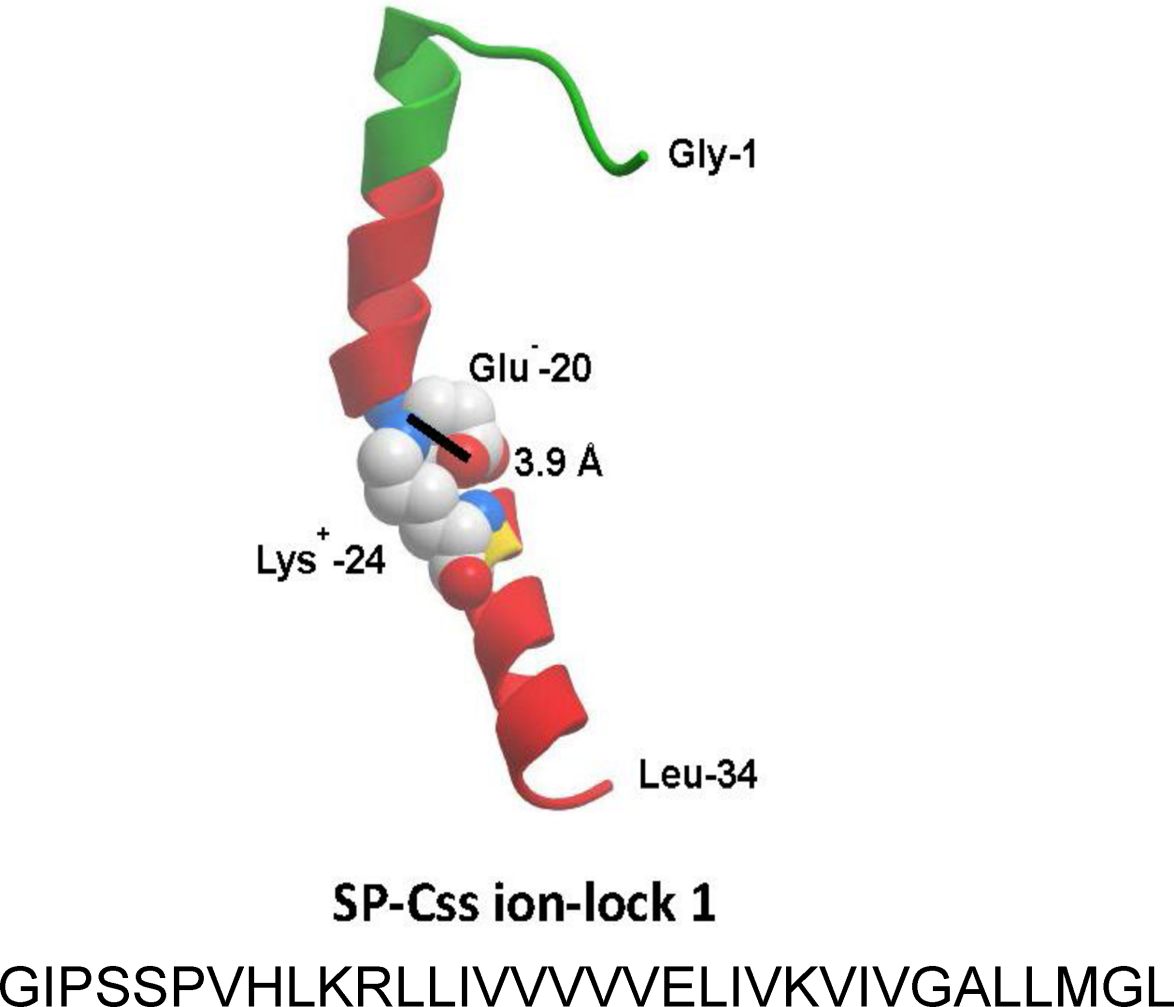
Linear sequence and homology-modeled ribbon structure of SP-Css ion-lock 1 peptide. The homology modeled structure of the active SP-Css ion-lock 1 peptide is shown with its primary helical motif in green and red ribbon, along with molecular modeling of the stabilizing salt bridge residues (Glu^−^-20, Lys^+^-24) and their equilibrium separation distance (3.9 Å) (Walther et al., 2014). The peptide is a 34 residue mimic of human SP-C that incorporates its primary active alpha helical residues with a stabilizing salt bridge (‘ion-lock’) linkage via residues Glu^−^-20 and Lys^+^-24. Two serine residues have also been substituted for cysteine residues in the N-terminal region of SP-Css ion-lock 1 compared to human SP-C to help reduce the tendency for aggregate formation.

The homology-modeled structures and linear sequences of S-MB DATK and SP-Css ion-lock 1 peptides are shown in Figs. 1 and 2. Both peptides were synthesized on a Symphony Multiple Peptide Synthesizer (Protein Technologies, Tucson, AZ) using a FastMoc™ protocol on a H-Ser(OtBu)-HMPB NovaPEG resin (Walther et al., 2010; Walther et al., 2014; Notter, Wang & Walther, 2016). All residues were double coupled to the resin for optimal yield. Crude peptides were cleaved from the resin using the standard phenol:thioanisole:ethanedithiol:water:trifluoroacetic acid (0.75:0.25:0.5:0.5:10 v:v) cleavage-deprotection mixture (Walther et al., 2010) and purified further (>95%) by preparative HPLC using a VYDAC diphenyl or C8 (1^′′^ by 12^′′^ width by length) column at 20 ml/min. Purified S-MB DATK and SP-Css ion-lock 1 were eluted from the column over one hour using a 0 to 100% linear gradient (water to acetonitrile with 0.1% TFA as an ion pairing agent added to both aqueous and organic phases). Because of the enhanced peptide molecular stability imparted by the designer-turn DATK substitution in S-MB DATK and the E20-K24 ion-lock in SP-Css ion-lock 1, further treatment to promote peptide folding and oxidation was not required. Purified peptide products were freeze-dried directly and their mass was confirmed by MALDI-TOF mass spectrometry.

**Figure 3:**
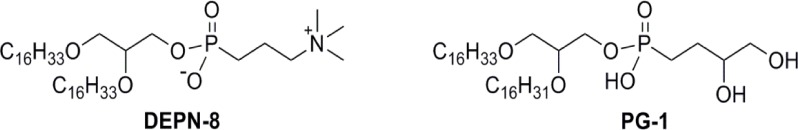
Schematic structures of phospholipase-resistant phosphonolipid analogs DEPN-8 and PG-1. DEPN-8 is a disaturated diether phosphonolipid analog of DPPC (C16:0, C16:0), while PG-1 is a mono-unsaturated diether phosphonolipid analog of POPG (C16:0, C16:1). The presence of ether rather than ester linkages at the chain-backbone junctions of DEPN-8 and PG-1, together with their headgroup phosphonate rather than phosphate moiety, make these analog compounds inherently structurally-resistant to chemical degradation by phospholipases A_1_, A_2_, and D. DEPN-8 is also partially resistant to phospholipase C due to steric hindrance. See text for details.

### Phospholipase-resistant lipids (DEPN-8, PG-1)

Phospholipase-resistant lipids used in synthetic surfactant mixtures were DEPN-8 and C16:0, C16:1 phosphono-PG (PG-1) (Fig. 3). Both lipids are structurally resistant to cleavage by phospholipases A_1_, A_2_ and D due to the presence of ether rather than ester linkages for the fatty chains plus a phosphono rather than phosphate moiety in the molecular headgroup. DEPN-8 [(±)-trimethyl(3-phosphonopropyl)ammonium, mono(2,3-bis(hexadecyloxy)propyl) ester] was synthesized and purified using previously reported methods (Turcotte et al., 1991; Wang et al., 2003; Notter et al., 2007; Wang et al., 2007). The chemical scheme for preparing DEPN-8 initially converted (±)-1-hexadecyloxy-2,3-propanediol to (±)-2,3-bis(hexadecyloxy)-1-propanol by way of hydroxyl protection at the 3-position, followed by alkylation at the 2-hydroxyl group and deprotection. Phosphonocholine placement involved treatment of (±)-2,3-bis(hexadecyloxy)-1-propanol with 3-bromopropylphosphono-dichloridic acid prepared from 3-bromopropylphosphonic acid and PCl_5_ (Wang et al., 2003), followed by reaction with Me_3_N in CHCl_3_:MeOH:H_2_O (10:10:1). After concentration, crude DEPN-8 was purified by exposure to Amberlite^®^, flash chromatography with elution by CHCl_3_:MeOH:H_2_O (60:35:5), and recrystallization from CHCl_3_/acetone. PG-1 [mono[2-[Z-9-hexadecen-1-yloxy]-3-(hexadecyloxy)propyl] P-(3,4-dihydroxybutyl) phosphonate] was also prepared based on our prior methods (Notter et al., 2007; Schwan et al., 2011). Dimethyl 3,4-bis(benzoyloxy)butylphosphonate was initially subjected to demethylative silylation of methoxy groups using TMS-Br, and the resulting bis(silylated) phosphonate was then directly converted to the analogous dichloride using oxalyl chloride. The dichloride was reacted without purification with (±)-2-[(Z)-9-hexadecen-1-yloxy]-3-(hexadecyloxy)–propanol to prepare a dibenzoyl-protected form of the phosphonolipid target. PG-1 was then obtained from this penultimate phosphonolipid through careful methanolysis of only the carboxylic ester functionalities while leaving the phosphonate ester intact. PG-1 was further purified by silica gel flash chromatography. The structures of DEPN-8 and PG-1 were verified by high-resolution mass spectrometry and by ^1^H and ^13^C NMR spectroscopy.

### Synthetic glycerophospholipids (5:3:2 DPPC:POPC:POPG)

DPPC, palmitoyl-oleoyl phosphatidylcholine (POPC), and POPG were obtained from Avanti Polar Lipids (Alabaster, AL, USA) at >99% purity.

### Synthetic lipid/peptide surfactant formulations

Synthetic surfactants were formulated to contain 35 mg/ml of either 9:1 DEPN-8:PG-1 or 5:3:2 DPPC:POPC:POPG combined with either 1.5% (by wt) S-MB DATK + 1.5% SP-Css ion-lock 1 together or 3% (by wt) of either peptide alone. Synthetic surfactant components were combined in organic solvent (e.g., chloroform for lipids and TFE for peptides), dried under nitrogen, exposed to house vacuum to remove residual solvent, resuspended by hand vortexing in 0.15 M NaCl (normal saline) adjusted to pH 7.0 with 0.1 N sodium bicarbonate, heated to 65 °C intermittently for 30 min, and refrigerated for >12 hours prior to use.

### Adsorption methods

Adsorption experiments were done at 37 °C in a Teflon dish with a 35 ml subphase (0.15 M NaCl, pH 7.0) stirred to minimize diffusion resistance (Notter, 2000; Notter, Wang & Walther, 2016). A bolus of surfactant containing 2.5 mg lipid in 5 ml of 0.15 M NaCl with pH 7.0 was injected into the stirred subphase, and adsorption surface pressure (surface tension lowering below that of the pure subphase) was measured as a function of time by the force on a partially submerged, sandblasted platinum Wilhelmy slide. The final surfactant phospholipid concentration for adsorption studies was uniform at 0.0625 mg/ml (2.5 mg surfactant phospholipid/40 ml of final subphase).

### Bubble surfactometer methods

The surface tension lowering ability of synthetic surfactants under dynamic compression at 37 °C was assessed by both pulsating and captive bubble surfactometers. The pulsating bubble surfactometer (General Transco Inc, Seminole, FL, USA) used was based on the design of Enhorning (Enhorning, 1977), while the captive bubble surfactometer was a fully-computerized version of that originally detailed by Schurch and co-workers (Schurch et al., 1989; Schurch et al., 1992). Measurements of overall surface activity with both instruments reflected a physiologically-relevant combination of adsorption and dynamic film compression at 20 cycles/min and body temperature (37 °C). Specific experimental procedures followed in dynamic surface activity studies were as reported previously by our group for the pulsating bubble surfactometer (Hall et al., 1993; Wang et al., 2003; Notter, Wang & Walther, 2016) and the captive bubble surfactometer (Walther et al., 2010). Pulsating bubble studies used an area compression ratio (maximum to minimum surface area) of 2:1 and were done at a uniform surfactant phospholipid concentration of 5 mg/ml (Notter, Wang & Walther, 2016). Captive bubble studies used a higher area compression ratio of 5:1, and were done at an average surfactant phospholipid concentration of 0.050 mg/ml (Walther et al., 2010).

### Shear viscosity measurements

Shear viscosities of synthetic surfactants were measured with a Wells–Brookfield cone and plate micro-viscometer (Model DV-II+; Brookfield Engineering Laboratories, Inc., Stoughton, MA) as detailed previously (King et al., 2001; King et al., 2002). Shear rate was varied by using one of two different spindles in the viscometer depending on desired torque and shear (CPE-40: 1–1500 s^−1^; CPE-51: 1–768 s^−1^). Before each experiment, the desired spindle and sample cup (CPE-44PSY) were washed with 95% ethanol and rinsed with nanopure water, and spindle position was calibrated to a gap height of 0.0005 inches above the sample cup. Surfactants for viscosity studies were dispersed in 0.15 M NaCl adjusted to pH 7 with sodium bicarbonate. A surfactant sample (0.5 ml volume at room temperature) was placed in the center of the viscometer sample cup using a syringe, and 10 min was allowed for temperature to equilibrate with a circulating water bath (37 °C). Viscosity was measured proceeding from low to high shear rates to minimize variability from shear-induced aggregate changes (King et al., 2001; King et al., 2002). Viscosity values at fixed shear rate were considered to be at a steady state after 15 s with no significant change. Clinical surfactants Infasurf™, Survanta™, and Curosurf™ for viscosity comparisons were obtained from the pharmacy of Strong Memorial Hospital, Rochester, NY, and used as formulated.

### Ventilated, lung-lavaged, surfactant-deficient rat and rabbit models

The preclinical pulmonary activity of synthetic surfactants was defined in studies in ventilated rats (phospholipase-resistant surfactants) and rabbits (glycerophospholipid surfactants) with surfactant deficiency/dysfunction induced by *in vivo* lung lavage. All animal studies were reviewed and approved by the Institutional Animal Care and Use Committee of the Los Angeles Biomedical Research Institute at Harbor-UCLA Medical Center (Research Projects # 12958 and 13230). All procedures and anesthesia were in accordance with the American Veterinary Medical Association (AMVA) Guidelines. Detailed methods used for anesthesia, surgery, lavage, ventilation, and animal monitoring have been reported in our prior work for rats (Walther et al., 2005; Walther et al., 2010) and rabbits (Walther et al., 2014; Notter, Wang & Walther, 2016).

In brief, rat studies utilized adult male Sprague-Dawley rats weighing 200–225 g. Rats were anesthetized, intubated, mechanically ventilated with 100% oxygen using a rodent ventilator, paralyzed, and an arterial line was placed for monitoring of blood pressure and blood gases. Airway pressures and tidal volumes were recorded continuously. Rats were gently lavaged with 0.9% NaCl to achieve a stable value of <100 mmHg for the arterial partial pressure of oxygen (arterial PO_2_), consistent with clinical ARDS (Bernard et al., 1994; Artigas et al., 1998). A dose of 100 mg/kg of a phospholipase-resistant synthetic surfactant or lipidonly controls was then instilled intratracheally, and arterial blood gases, tidal volume and airway pressures were determined at 15 min intervals for the remainder of study. Dynamic lung compliance was calculated by dividing tidal volume/kg body weight by changes in airway pressure (peak inspiratory pressure minus positive end-expiratory pressure) (mL/kg/cmH_2_O). Rats were euthanized 90 min after surfactant instillation, and each treatment group consisted of 8–10 animals. In analogous rabbit studies, young adult New Zealand white rabbits (weight 1.0–1.3 kg) were anesthetized and a venous line was placed via a marginal ear vein. Rabbits were intubated, received a carotid arterial line, and were placed on a Harvard volume-controlled animal ventilator with 100% oxygen. After stabilization on the ventilator under paralysis, saline lavage was performed until arterial PO_2_ was stable at <100 mmHg, and rabbits were then given 100 mg/kg of glycerophospholipid synthetic surfactant or lipid-only controls by intratracheal instillation. Arterial pH and blood gases and dynamic lung compliance were subsequently obtained every 15 min until the end of study. Rabbits were euthanized 120 min after surfactant administration, and each treatment group consisted of 5–9 animals.

### Statistical analysis

All data are expressed as Mean ± 1 S.E.M. Statistical analyses used Student’s *t*-test for comparisons of discrete data points, and functional data were analyzed by one-way analysis of variance (ANOVA) with Scheffe’s *post hoc* analysis to adjust for multiple comparisons. Differences were considered statistically significant if the probability of the null hypothesis (P) was <0.05 or less.

## Results

### Adsorption activity of synthetic lipid/peptide surfactants

All the synthetic lipid/peptide surfactants studied had substantial activity in adsorbing rapidly to the air-water interface to generate high surface pressures (low adsorption surface tensions). Synthetic surfactants containing 9:1 DEPN-8:PG-1 or 5:3:2 DPPC:POPC:POPG combined with 1.5% S-MB DATK + 1.5% SP-Css ion-lock 1 peptides together, or with 3% S-MB DATK alone, had the greatest adsorption activity. These surfactants adsorbed rapidly to surface pressures of 43–46 mN/m (surface tensions of 24–27 mN/m at 37 °C) within 60 s, and had final equilibrium surface pressure values of 46–48 mN/m (equilibrium surface tensions of 22–24 mN/m at 37 °C). Equilibrium adsorption surface pressures of this magnitude are very close to the interfacial spreading limit of fluid phospholipids at body temperature. This very fast adsorption was present despite the low surfactant phospholipid concentration studied (0.0625 mg/ml, see Methods). Synthetic lipid/peptide surfactants containing 9:1 DEPN-8:PG-1 or 5:3:2 DPPC:POPC:POPG combined with 3% SP-Css ion-lock 1 peptide alone had lower but still substantial adsorption facility, reaching surface pressures of ∼36 mN/m after 60 sec and ∼44 mN/m at final equilibrium (equivalent to adsorption surface tensions of 34 and 26 mN/m, respectively, at 37 °C).

### Dynamic surface activity of synthetic lipid/peptide surfactants

**Figure 4:**
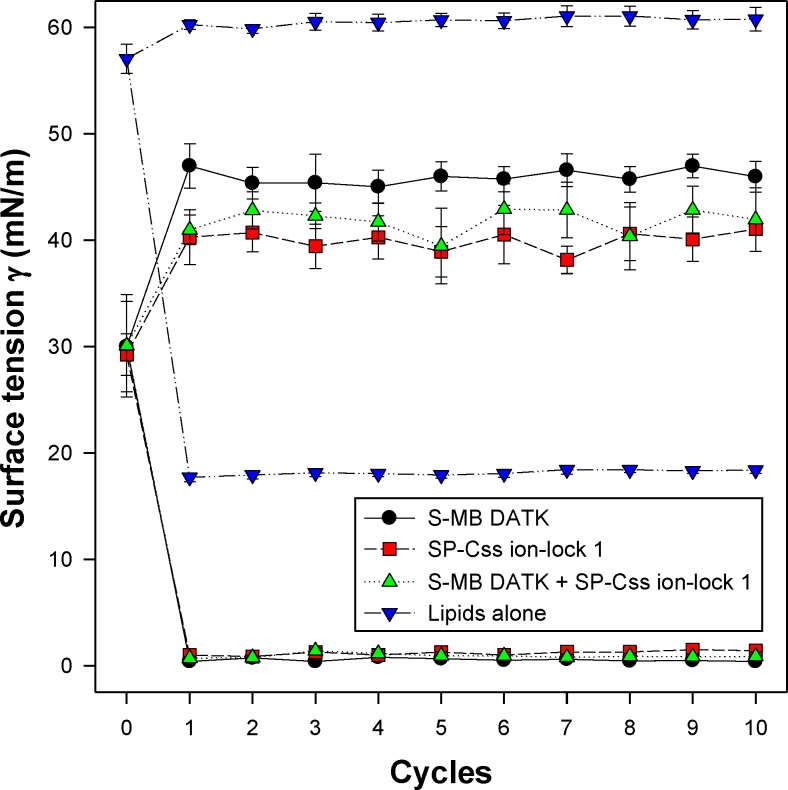
Captive bubble activity of synthetic surfactants containing glycerophospholipids plus SP-B/C peptides. Dynamic surface activity of synthetic lipid/peptide lung surfactants was studied with a captive bubble surfactometer at 37°C. Synthetic surfactants studied were 5:3:2 (weight ratio) DPPC:POPC:POPG (lipids alone) combined with 1.5% S-MB DATK + 1.5% SP-Css ion-lock 1 peptides together or with 3% of either peptide alone. Each sample includes two plots, the lower plot represents minimum surface tension and the upper represents maximum surface tension. Synthetic glycerophospholipid/peptide surfactants were all highly active in reducing minimum surface tension to ≤1 mN/m on the captive bubble surfactometer throughout 10 consecutive compression-expansion cycles. See text for details. Data are Mean ± S.E.M. for *N* = 4–8.

**Figure 5:**
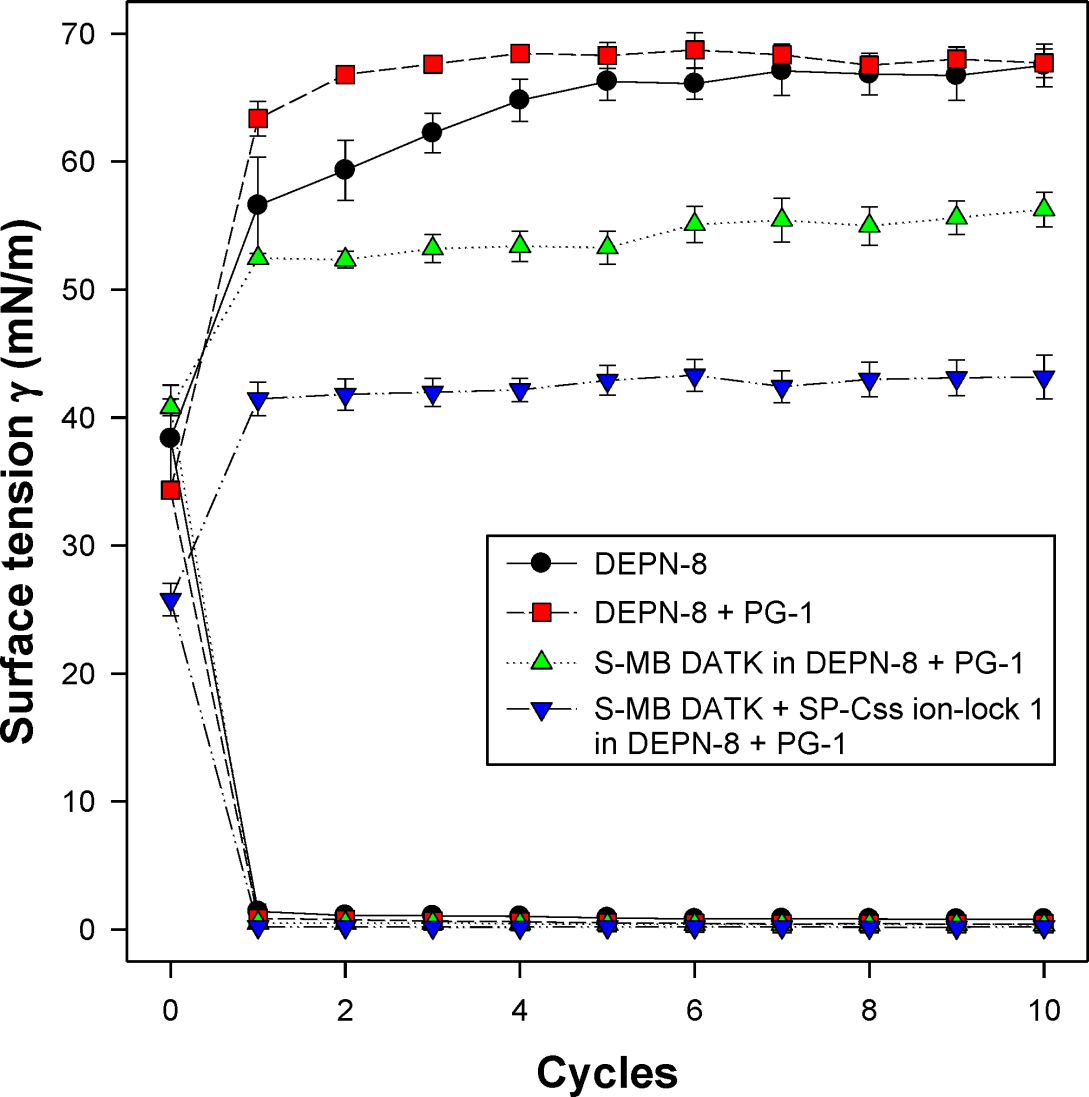
Captive bubble activity of synthetic lung surfactants containing phospholipase-resistant lipids plus SP-B/C peptides. Phospholipase-resistant synthetic surfactants containing 9:1 DEPN-8:PG-1 combined with 1.5% S-MB DATK + 1.5% SP-Css ion-lock 1 peptides together, or 3% of S-MB DATK alone, were studied for dynamic surface tension lowering activity on a captive bubble surfactometer as in Fig. 4. Each sample includes two plots, the lower plot represents minimum surface tension and the upper plot represents maximum surface tension. The phospholipase-resistant synthetic lipid/peptide surfactants were highly active in reducing minimum surface tension to values ≤1 mN/m on the captive bubble surfactometer. In addition, DEPN-8 and 9:1 DEPN-8:PG-1 also reached low minimum surface tensions on the bubble due to improved adsorption relative to glycerophospholipids. See text for details. Data are Mean ± S.E.M. for *N* = 3–4.

All synthetic surfactants containing 9:1 DEPN-8:PG-1 or 5:3:2 DPPC:POPC:POPG combined with 1.5% S-MB DATK + 1.5% SP-Css ion-lock 1 peptides together, or 3% of either peptide alone, had very high activity in reducing minimum surface tension to <1 mN/m during dynamic cycling in both pulsating and captive bubble studies. Dynamic surface tension lowering data for the first ten cycles of compression/expansion on the captive bubble surfactometer are shown in Fig. 4 (glycerophospholipid surfactants) and Fig. 5 (phospholipase-resistant surfactants). All the synthetic lipid/peptide surfactants studied generated extremely low surface tension values of <1 mN/m on the first compression cycle, which was initiated following a five minute pause to allow adsorption after surfactant was first deposited in the captive bubble sample chamber. A control mixture of glycerophospholipids alone (5:3:2 DPPC:POPC:POPG) had low surface activity on the captive bubble (Fig. 4), while phospholipase-resistant lipid-only controls (DEPN-8 and 9:1 DEPN-8:PG-1) were able to reduce surface tension to <1 mN/m even in the absence of peptides (Fig. 5). This latter finding presumptively reflects the previously reported high adsorption of DEPN-8 relative to glycerophospholipids (Turcotte et al., 1991; Wang et al., 2003), which would lead to a greater surface film concentration (at fixed subphase concentration) for this resistant lipid in bubble studies.

### *In vivo* studies in surfactant-deficient rat and rabbit models

**Figure 6:**
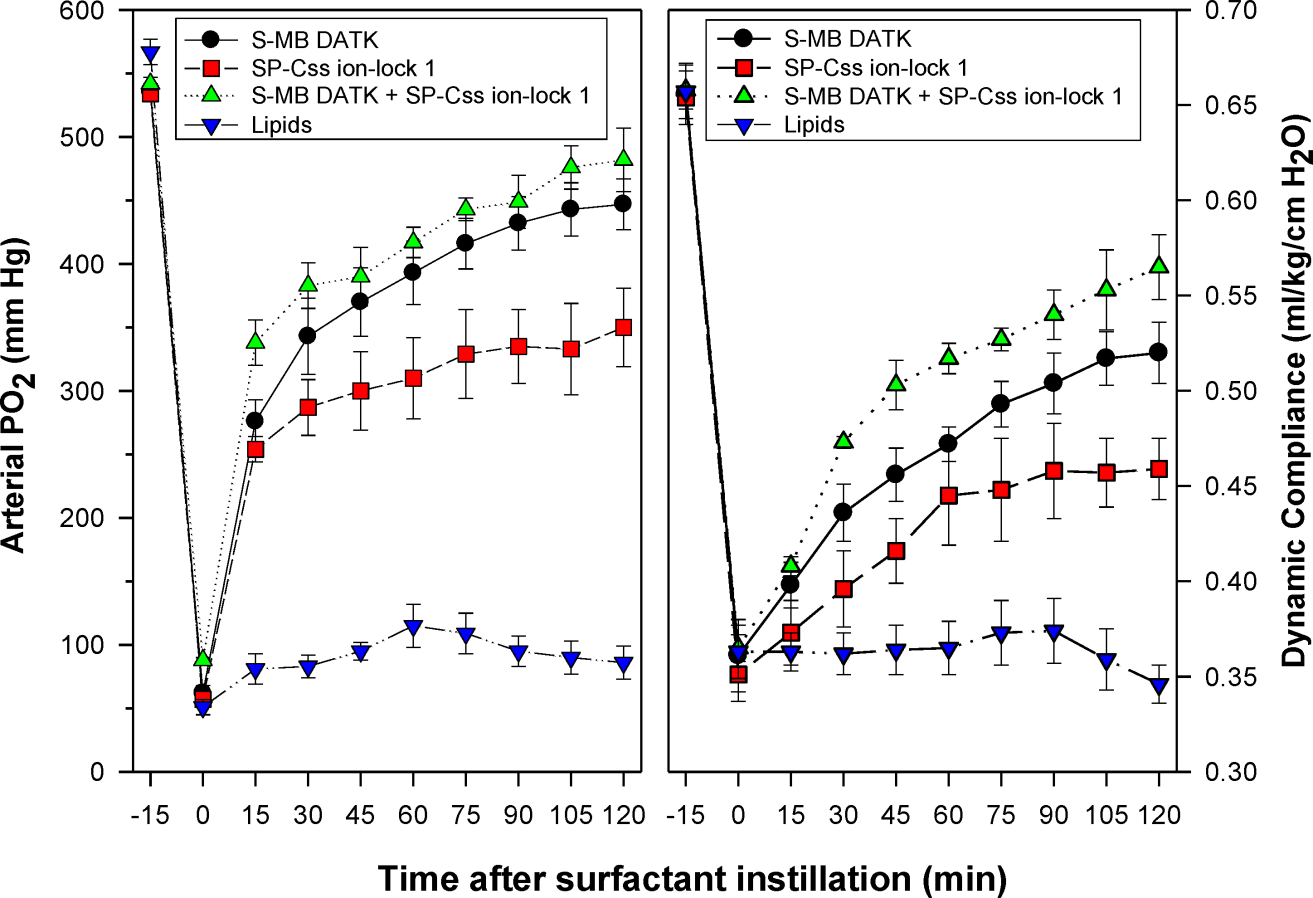
Physiological activity of synthetic surfactants containing glycerophospholipids and SP-B/C peptides in ventilated rabbits with ARDS-related lung injury induced by *in vivo* lavage. Synthetic surfactants containing lipids (5:3:2 DPPC:POPC:POPG) combined with 1.5% S-MB DATK + 1.5% SP-Css ion-lock 1 peptides or 3% of either peptide alone, were instilled intratracheally to rabbits meeting clinical oxygenation criteria for ARDS. All glycerophospholipid/peptide surfactants significantly improved oxygenation (arterial PO_2_) and dynamic compliance (mL/kg/cm H_2_O) in rabbits over a study period of 120 min compared to lipid-only controls (*P* < 0.05). However, the greatest pulmonary improvements were found for the dual-peptide surfactant preparation containing 1.5% S-MB DATK + 1.5% SP-Css ion-lock 1 (*P* < 0.05 for both oxygenation and dynamic compliance compared to either single-peptide surfactant). Data are Mean ± S.E.M. for *N* = 5–9 per treatment group.

**Figure 7:**
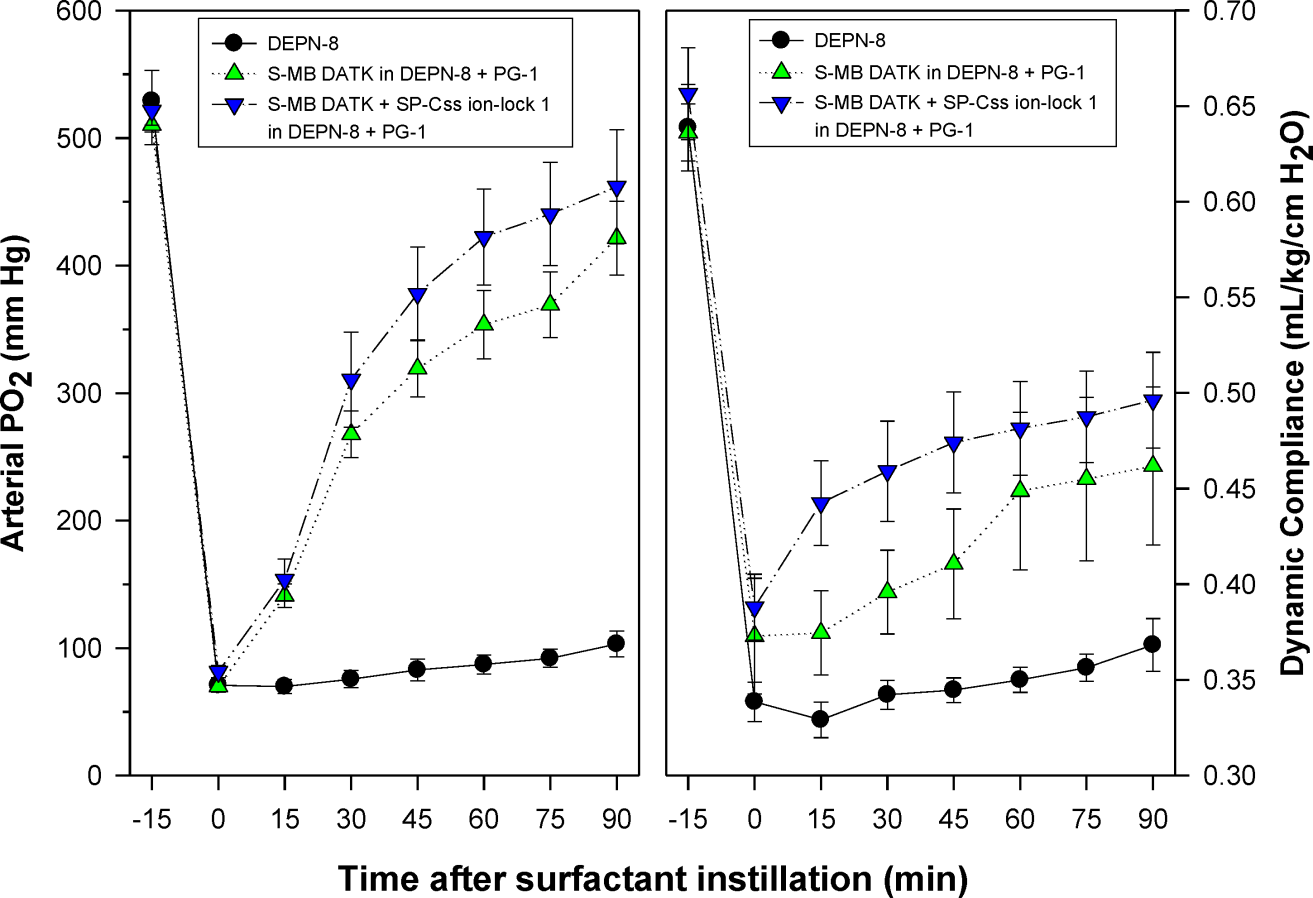
Physiological activity of phospholipase-resistant synthetic surfactants with SP-B/C peptides in ventilated rats with ARDS-related lung injury induced by *in vivo* lavage. Phospholipase-resistant synthetic surfactants were instilled intratracheally in rats meeting clinical oxygenation criteria for ARDS, and arterial oxygenation (arterial PO_2_) and dynamic lung compliance (mL/kg/cm H_2_O) were then measured over a study period of 90 min to assess surfactant efficacy. Single peptide phospholipase-resistant synthetic surfactants containing 3% S-MB DATK had significant pulmonary activity compared to lipid control (*P* < 0.05). The dual-peptide surfactant containing 1.5% S-MB DATK + 1.5% SP-Css ion-lock 1 showed further improvement in arterial oxygenation and dynamic compliance compared to the surfactant containing 3% S-MB DATK alone (*P* < 0.05). Data are Mean ± S.E.M. for *N* = 7–8 per treatment group.

Synthetic surfactants were administered to ventilated animals by intratracheal instillation after the arterial partial pressure of oxygenation was reduced by *in vivo* saline lavages to stable levels of <100 mm Hg when breathing 100% oxygen, well-below the threshold clinical criteria for ARDS (Bernard et al., 1994). All synthetic lipid/peptide surfactants had significant pulmonary activity compared to lipid-only controls when administered intratracheally to rabbits (glycerophospholipid surfactants) or rats (phospholipase-resistant lipid surfactants) with ARDS (Figs. 6 and 7, *P* < 0.05 by one-way ANOVA compared to control). However, although all synthetic lipid/peptide surfactants had significant pulmonary activity compared to lipid-only controls, dual-peptide surfactants containing either glycerophospholipids or phospholipase-resistant lipids combined with 1.5% S-MB DATK + 1.5% SP-Css ion-lock 1 had greater activity than single-peptide surfactants in improving both arterial oxygenation and dynamic lung compliance (*P* < 0.05 by one-way ANOVA). For synthetic glycerophospholipid/peptide surfactants (Fig. 6), comparative single-peptide surfactants contained either 3% S-MB DATK or 3% SP-Css ion-lock 1. For phospholipase-resistant lipid/peptide surfactants (Fig. 7), the comparative single-peptide surfactant contained 3% S-MB DATK, since it had greater activity than 3% SP-Css ion-lock 1 in studies with glycerophospholipid surfactants in Fig. 6.

### Shear viscosities of synthetic surfactant preparations

Shear viscosities of saline dispersions of synthetic lipid/ peptide surfactants were measured to help assess their potential ease of deliverability via tracheal instillation in future clinical applications. Viscosity results were defined as a function of shear rate for phospholipase-resistant lipid/peptide surfactants (Table 1) and glycerophospholipid/peptide surfactants (Table 2). Comparative viscosity measurements were also made for clinical formulations of current animal-derived surfactant drugs (Table 1). All synthetic and animal-derived surfactants studied exhibited non-Newtonian rheological behavior with higher viscosities at lower shear rates. However, dual-peptide synthetic surfactants containing phospholipase-resistant lipids or glycerophospholipids combined with 1.5% S-MB DATK + 1.5% SP-Css ion-lock 1 had equal or lower viscosity values than current clinical formulations of animal-derived surfactant drugs across the range of shear rates studied (Tables 1 and 2). Synthetic surfactants containing 3% S-MB DATK had comparable or lower viscosity values compared to dual-peptide synthetic surfactants, while preparations containing 3% SP-Css ion-lock 1 exhibited somewhat larger viscosities particularly at low shear rate.

**Table 1:** Shear viscosities of phospholipase-resistant synthetic surfactants containing SP-B/C peptides. Viscosities measured as a function of shear rate are shown for synthetic surfactant suspensions containing 9:1 DEPN-8:PG-1 combined with either 3% (by wt) S-MB DATK, 3% SP-Css ion-lock 1, or 1.5% S-MB DATK + 1.5% SP-Css ion-lock 1 peptides. Viscosities of selected current clinical animal-derived surfactant drugs as formulated are shown for comparison.

Viscosity (Centipoise) at Different Shear Rates (S^−1^)									
Surfactants	33	77	225	154	230	307	384	576	768
DEPN-8	4.7 ± 0.5	3.9 ± 0.5	3.2 ± 0.1	2.8 ± 0.3	2.7 ± 0.2	2.6 ± 0.2	2.3 ± 0.1	2.3 ± 0.1	2.3 ± 0.1
DEPN-8:PG-1	4.2 ± 0.3	3.7 ± 0.4	3.4 ± 0.2	3.1 ± 0.3	2.8 ± 0.1	2.8 ± 0.1	2.6 ± 0.2	2.5 ± 0.1	2.5 ± 0.1
DEPN-8:PG (9:1) + 3% S-MB DATK									
	16.7 ± 1.9	14.7 ± 1.2	11.0 ± 1.7	9.2 ± 1.7	7.6 ± 1.3	5.9 ± 0.9	4.5 ± 0.9	4.1 ± 0.9	3.8 ± 0.8
DEPN-8:PG (9:1) + 3% SP-Css ion-lock 1									
	30.2 ± 2.1	26.5 ± 2.3	20.5 ± 0.8	17.2 ± 1.1	14.4 ± 0.8	10.4 ± 1.2	8.9 ± 1.1	8.0 ± 1.5	6.2 ± 1.7
DEPN-8:PG (9:1) + 1.5% S-MB DATK + 1.5% SP-Css ion-lock 1									
	18.7 ± 2.6	16.0 ± 2.1	11.8 ± 2.3	9.8 ± 2.6	7.9 ± 1.6	6.1 ± 2.0	4.4 ± 1.3	4.0 ± 1.4	3.6 ± 1.4
Infasurf	26.9 ± 0.9	25.0 ± 0.4	19.3 ± 0.3	15.8 ± 0.2	12.4 ± 0.2	10.4 ± 0.2	9.1 ± 0.1	7.5 ± 0.1	6.6 ± 0.1
Survanta	34.2 ± 2.1	28.8 ± 0.4	21.1 ± 0.8	18.8 ± 0.7	15.6 ± 0.4	13.7 ± 0.2	12.2 ± 0.6	9.7 ± 0.1	9.1 ± 0.1
Curosurf	19.5 ± 1.2	14.1 ± 2.0	12.6 ± 1.8	11.6 ± 1.0	10.4 ± 1.4	9.7 ± 1.2	9.2 ± 1.2	8.5 ± 1.1	8.3 ± 1.0

**Notes.**

See Methods for experimental details. Concentrations of synthetic phospholipase-resistant surfactants were 35 mg lipid/ml, and samples of animal-derived clinical surfactants were as clinically formulated, i.e., Infasurf (35 mg/ml), Survanta (25 mg/ml), and Curosurf (80 mg/ml). All data are Mean ± S.E.M. for *N* = 4.

**Table 2:** Shear rate viscosities of synthetic glycerophospholipid:peptide surfactants as a function of shear rate. Viscosities measured as a function of shear rate are shown for synthetic lung surfactants containing glycerophospholipids (5:3:2 DPPC:POPC:POPG) combined with either 3% S-MB DATK, 3% SP-Css ion-lock 1, or 1.5% S-MB DATK + 1.5% SP-Css ion-lock 1 peptides.

Viscosity (Centipoise) at Different Shear Rates (S^−1^)									
Surfactants	33	77	225	154	230	307	384	576	768
5:3:2 DPPC:POPC:POPG									
35 mg/ml	2.6 ± 0.5	1.7 ± 0.2	1.6 ± 0.1	1.5 ± 0.1	1.4 ± 0.1	1.4 ± 0.1	1.4 ± 0.1	1.4 ± 0.1	1.4 ± 0.1
5:3:2 DPPC:POPC:POPG + 3% S-MB DATK									
35 mg/ml	5.0 ± 0.2	4.3 ± 0.4	3.9 ± 0.2	3.7 ± 0.2	3.6 ± 0.2	3.5 ± 0.2	3.3 ± 0.2	3.1 ± 0.2	3.0 ± 0.1
60 mg/ml	10.6 ± 0.4	9.4 ± 0.8	8.8 ± 0.8	8.1 ± 0.8	7.7 ± 0.8	7.1 ± 0.7	6.8 ±0.7	6.4 ± 0.6	6.0 ± 0.6
5:3:2 DPPC:POPC:POPG + 3% SP-Css ion-lock 1									
35 mg/ml	31.4 ± 3.3	26.4 ± 2.2	23.0 ± 3.1	19.5 ± 3.4	16.4 ± 2.9	13.9 ± 2.5	11.7 ± 2.0	9.7 ± 1.6	7.7 ± 1.2
60 mg/ml	42.2 ± 4.9	36.9 ± 5.4	30.5 ± 4.6	28.6 ± 4.0	19.9 ± 1.4	18.8 ± 1.7	16.8 ± 1.7	13.5 ± 3.5	11.5 ± 2.7
5:3:2 DPPC:POPC:POPG + 1.5% S-MB DATK + 1.5% SP-Css ion-lock 1									
35 mg/ml	6.0 ± 0.4	4.7 ± 0.5	4.2 ± 0.6	4.0 ± 0.6	3.7 ± 0.6	3.6 ± 0.6	3.5 ± 0.6	3.3 ± 0.6	3.2 ± 0.6
60 mg/ml	12.7 ± 0.5	11.8 ± 0.3	10.5 ± 0.3	9.2 ± 0.2	8.8 ± 0.2	7.9 ± 0.2	7.5 ± 0.4	6.7 ± 0.5	6.3 ± 0.6

**Notes.**

See Methods for experimental details, and Table 1 for the viscosities of selected animal-derived clinical surfactants. Data are Mean ± S.E.M. for *N* = 3–4.

## Discussion

This paper has investigated the *in vitro* surface activity, *in vivo* pulmonary efficacy, and shear viscosity of synthetic lung surfactants containing novel phospholipase-resistant lipids (9:1 DEPN-8/PG-1) or synthetic glycerophospholipids (5:3:2 DPPC:POPC:POPG) combined with S-MB DATK and SP-Css ion-lock 1 peptides bioengineered to replicate the major functional activities of human surfactant proteins SP-B and SP-C. Extensive prior *in silico* research on the structure and stability of these synthetic peptides has documented their ability to achieve functional molecular biophysical interactions with lipids analogous to the parent human proteins (Walther et al., 2014; Notter, Wang & Walther, 2016). In addition, both peptides incorporate focused residue substitutions to enhance structural stability and ease of synthesis/purification as pharmaceuticals. Specifically, S-MB DATK contains a DATK designer loop substitution to enhance the stability of the saposin fold domain that is functionally critical in native SP-B (Notter, Wang & Walther, 2016), while SP-Css ion-lock 1 contains an electro-neutral salt-bridge or ‘ion-lock’ pair (E20-K24) to stabilize against detrimental non-specific beta (amyloid-like) structural formation that can compromise the activity of native SP-C (Walther et al., 2014) (Figs. 1 and 2).

Results here showed that synthetic surfactants containing either phospholipase-resistant lipids or glycerophospholipids combined with S-MB DATK or SP-Css ion-lock 1 peptide alone had substantial surface and pulmonary activity (Figs. 6 and 7), in agreement with our recent positive reports on single-peptide glycerophospholipid synthetic surfactants (Walther et al., 2014; Notter, Wang & Walther, 2016). Studies here also showed that single-peptide glycerophospholipid surfactants containing 3% S-MB DATK had higher pulmonary activity than those containing 3% SP-Css ion-lock 1 (Fig. 6), consistent with prior work showing that native SP-B has particularly powerful biophysical interactions with phospholipids to promote functional activity in lung surfactants (Curstedt et al., 1987; Notter, 2000; Notter et al., 2002; Wang et al., 2002). However, an important added finding for both phospholipase-resistant and glycerophospholipid synthetic surfactants was that maximum pulmonary activity was exhibited by preparations containing both peptides together (Figs. 6 and 7). This behavior is directly analogous to the known functional composition of native lung surfactant, which includes both SP-B and SP-C apoproteins as has been extensively documented in basic research (Notter & Wang, 1997; Creuwels, Van Golde & Haagsman, 1997; Notter, 2000). Both SP-B and SP-C are also present in the bovine surfactant extract CLSE that is the basis of the clinically-efficacious surfactant drug Infasurf™ (Wang, Hall & Notter, 1996; Notter, 2000; Notter et al., 2002), as well as in column-processed lung extracts that form the basis of the clinically-active porcine surfactant drug Curosurf™ (Curstedt et al., 1987; Robertson et al., 1990). An interesting issue is how to envision the “cooperation” between SP-B and SP-C analogs in a surfactant lipid mixture. Although both S-MB DATK and SP-Css ion-lock 1 effectively reduce surface tension and the combination further enhances surface activity, the main surface tension lowering effect is produced by the SP-B analog. This dominant SP-B effect might be explained by the recent finding that native SP-B forms higher-ordered ring structures composed of 5 or 6 covalent dimers that may profoundly influence surface properties (Olmeda, Garcia-Álvarez & Pérez-Gil, 2013; Olmeda et al., 2015).

In addition to defining the surface activity and preclinical pulmonary efficacy of dual-peptide synthetic surfactants, another specific emphasis in this study was to further the development of phospholipase-resistant synthetic surfactants of potential utility for targeting inflammatory lung injury and ARDS. Results were promising in showing that dual-peptide phospholipase-resistant synthetic surfactants containing 9:1 DEPN-8:PG-1 combined with 1.5% S-MB DATK + 1.5% SP-Css ion-lock 1 had high *in vitro* surface activity and *in vivo* pulmonary efficacy in ventilated rats with ARDS (Fig. 7). The phospholipase-resistant lipids DEPN-8 and PG-1 in this preparation have designed biophysical properties to promote high surface activity. DEPN-8 has close structural analogy to DPPC, the predominant disaturated phospholipid that allows interfacial films of endogenous surfactant to generate very low surface tensions under dynamic compression following apoprotein-mediated adsorption (Notter, 2000; Wang et al., 2003; Notter et al., 2007). Ether linkages between the fatty chains and glycerol backbone in DEPN-8 increase its molecular flexibility and hydrophobicity compared to ester-linked DPPC, with only a minimal steric penalty in packing ability (Turcotte et al., 1991; Skita et al., 1995). Thus, DEPN-8 not only has the ability to reduce surface tension to <1 mN/m in compressed interfacial films, but also has superior adsorption and film respreading compared to DPPC (Turcotte et al., 1991; Skita et al., 1995; Wang et al., 2003). DEPN-8 can also form interdigitated as well as normal opposed bilayers, which may contribute to its improved respreading and adsorption (Skita et al., 1995). The C16:0 fatty chains of DEPN-8 are also fully miscible with the C16:0 chain on mono-unsaturated PG-1 (C16:0, C16:1), facilitating lipid-lipid interactions in phospholipase-resistant surfactants (Schwan et al., 2011). In addition, the anionic PG-1 head group allows ionic interactions with positively charged residues on surfactant peptides. Although much is known about the biophysics of these pure non-hydrolysable phospholipid analogs, biocompatibility of these preparations has only received limited attention.

As noted earlier, current exogenous surfactant drugs all have a high content of phospholipids that are structurally sensitive to the chemical action of phospholipases that have been found in the pulmonary interstitium and alveoli during inflammatory injury (Touqui & Arbibe, 1999; Attalah et al., 2003; Nakos et al., 2003; Notter et al., 2007; Machado-Aranda et al., 2013). Phospholipase A_2_(PLA_2_) may have specific importance in the pathogenesis of ARDS (Touqui & Arbibe, 1999) and meconium-induced lung injury in term infants (Kaapa, 2001; Schrama et al., 2001). Phospholipases not only impair the composition and activity of lung surfactants (Holm et al., 1991; Schrama et al., 2001; Wang et al., 2007), but the chemical byproducts of lipid degradation such as lysophosphatidylcholine and fluid free fatty acids are themselves biophysical inhibitors of surfactant activity (Wang & Notter, 1998; Holm, Wang & Notter, 1999; Wang et al., 2005) and also cause permeability injury at the level of the alveolocapillary membrane (Niewoehner et al., 1987; Hall et al., 1990). The ability to construct synthetic lipid/peptide exogenous surfactants that resist phospholipase degradation while exhibiting high surface and pulmonary activity thus has potential benefits on a number of levels.

In addition to studying the activity of synthetic lipid/peptide surfactants, experiments here also measured the clinically-relevant shear viscosities of dispersions of synthetic lipid/peptide surfactants (Tables 1 and 2). The viscosity of surfactant drugs not only affects their ease of intratracheal or bronchoscopic instillation, but also the subsequent ability to transport through the pulmonary airways to reach their alveolar site of action. High viscosities impair this process. The non-Newtonian viscosities of native lung surfactant and animal-derived exogenous surfactants are known to be highest at low shear rates (e.g., <100 s^−1^) (King et al., 2001; King et al., 2002). Low shear rates in this range are directly applicable for surfactant distributional flows in small airways in the pulmonary periphery. Results here showed that synthetic lipid/peptide surfactant dispersions have non-Newtonian viscous behavior conceptually similar to animal-derived exogenous surfactants, i.e., viscosity magnitudes increased at lower shear rates (Tables 1 and 2). However, viscosity results were positive in documenting the ability to formulate synthetic lipid/peptide surfactants at clinically-relevant concentrations (35–60 mg/ml depending on composition) to have shear viscosities lower or equal to those of current clinical formulations of animal-derived surfactant drugs across a range of shear rates.

There is a significant need to develop improved surfactant therapy for clinical lung injury and ARDS. Fully synthetic surfactant drugs as defined here have multiple pharmacologic advantages over animal-derived drugs, and have the potential to favorably impact the economy of exogenous surfactant use in ARDS (Notter et al., 2007; Notter, Wang & Walther, 2016). Most importantly, the development of highly-active synthetic lipid/peptide surfactants as defined here, including both phospholipase-resistant and glycerophospholipid formulations, may help to improve the efficacy of future surfactant therapies for inflammatory direct lung injury and ARDS.

##  Supplemental Information

10.7717/peerj.2635/supp-1 Data S1 Captive bubble surfactometry measurements (pages 1 and 2) and in vivo lung function data (pages 3 and 4) Page 1 (Fig. 4) shows the captive bubble surfactometry (CBS) data of the first 10 quasi-static cycles of 3% S-MB DATK, 3% SP-Css ion-lock 1 or 1.5% S-MB DATK + 1.5% SP-Css ion-lock 1 in 35 mg/ml of DPPC:POPC:POPG 5:3:2 (wt:wt:wt) or DPPC:POPC:POPG 5:3:2 (wt:wt:wt) = lipids alone. Page 2 (Fig. 5) provides similar CBS data as Page 1 for 35 mg/ml of DEPN-8 (*n* = 4), 35 mg/ml of DEPN-8:PG-1 9:1 (wt:wt) (*n* = 4), and 3% S-MB DATK (*n* = 3) and 1.5% S-MB DATK + 1.5% SP-Css ion-lock 1 (*n* = 4) in 35 mg/ml of DEPN-8:PG-1 9:1 (wt:wt). Page 3 (Fig. 6) shows the lung function data (arterial oxygenation = PaO2 in mmHg and dynamic compliance in ml/kg/cm H2O) of 29 ventilated, lavaged, surfactant-deficient rabbits treated with 3% S-MB DATK (*n* = 9), 3% SP-Css ion-lock 1 (*n* = 6), or 1.5% S-MB DATK + 1.5% SP-Css ion-lock 1 (*n* = 5) in 35 mg/ml of DPPC:POPC:POPG 5:3:2 (wt:wt:wt) or DPPC:POPC:POPG 5:3:2 (wt:wt:wt) alone (*n* = 9). Page 4 (Fig. 7) shows the lung function data (arterial oxygenation = PaO2 in mmHg and dynamic compliance in ml/kg/cm H2O) of 23 ventilated, lavaged, surfactant-deficient rats treated at time 0 with phospholipase-resistant surfactants consisting of 35 mg/ml of DEPN-8:PG-1 9:1 (wt:wt) with 3% S-MB DATK (*n* = 8) or 1.5% S-MB DATK + 1.5% SP-Css ion-lock 1 (*n* = 7) or DEPN-8 alone (*n* = 8)

## Additional Information and Declarations

### Data Availability

The following information was supplied regarding data availability: The raw data have been supplied as a Data S1.
